# Need for awareness and surveillance of long-term post-COVID neurodegenerative disorders. A position paper from the neuroCOVID‐19 task force of the European Academy of Neurology

**DOI:** 10.1007/s00415-025-13110-3

**Published:** 2025-05-06

**Authors:** Dániel Bereczki, Ádám Dénes, Filippo M. Boneschi, Tamar Akhvlediani, Francesco Cavallieri, Alessandra Fanciulli, Saša R. Filipović, Alla Guekht, Raimund Helbok, Sonja Hochmeister, Tim J. von Oertzen, Serefnur Özturk, Alberto Priori, Martin Rakusa, Barbara Willekens, Elena Moro, Johann Sellner, on behalf of the NeuroCOVID-19 Task Force of the European Academy of Neurology

**Affiliations:** 1 Department of Neurology, Semmelweis University, Budapest, Hungary; 2 HUN-REN SU Neuroepidemiology Research Group, Budapest, Hungary; 3 “Momentum” Laboratory of Neuroimmunology, HUN-REN Institute of Experimental Medicine, Budapest, 1083 Hungary; 4 Clinical Neurology, Department of Health Science CRC “Aldo Ravelli” for Experimental Brain Therapeutics, University of Milan, Milan, Italy; 5 Hospital San Paolo, ASST Santi Paolo E Carlo, Milan, Italy; 6 Department of Neurology, Neurosurgery and Addiction Medicine, Georgian-American University, Tbilisi, Georgia; 7 Neurology Unit, Neuromotor and Rehabilitation Department, Azienda USL-IRCCS Di Reggio Emilia, Reggio Emilia, Italy; 8 Department of Neurology, Medical University of Innsbruck, Innsbruck, Austria; 9 Institute for Medical Research, University of Belgrade, Belgrade, Serbia; 10 Research and Clinical Center for Neuropsychiatry, Moscow, Russian Federation; 11 Pirogov Russian National Research Medical University, Moscow, Russian Federation; 12 Department of Neurology, Kepler University Hospital, Johannes Kepler University Linz, Linz, Austria; 13 Clinical Research Institute of Neuroscience, Johannes Kepler University Linz, Kepler University Hospital, Linz, Austria; 14 Department of Neurology, Medical University of Graz, Graz, Austria; 15 Medical Directorate, University Hospital Würzburg, Würzburg, Germany; 16 Department of Neurology, Faculty of Medicine, Selcuk University, Konya, Turkey; 17 Aldo Ravelli’ Centre for Neurotechnology and Experimental Brain Therapeutics, Department of Health Sciences, University of Milan, Milan, Italy; 18 Clinical Neurology Unit, Department of Health Sciences, ‘Azienda Socio-Sanitaria Territoriale Santi Paolo E Carlo’, University of Milan, Milan, Italy; 19 Division of Neurology, University Medical Centre Maribor, Maribor, Slovenia; 20 Department of Neurology, Antwerp University Hospital, Edegem, Belgium; 21 Translational Neurosciences Research Group, University of Antwerp, Wilrijk, Belgium; 22 Division of Neurology, CHU of Grenoble, Grenoble Institute of Neurosciences, INSERM U1216, Grenoble Alpes University, Grenoble, France; 23 Department of Neurology, Landesklinikum Mistelbach-Gänserndorf, Mistelbach, Austria

**Keywords:** Awareness, Surveillance, Inflammation, Neurodegeneration, SARS-CoV-2

## Abstract

**Background:**

Neuropathological and clinical studies suggest that infection with SARS-CoV-2 may increase the long-term risk of neurodegeneration.

**Methods:**

We provide a narrative overview of pathological and clinical observations justifying the implementation of a surveillance program to monitor changes in the incidence of neurodegenerative disorders in the years after COVID-19.

**Results:**

Autopsy studies revealed diverse changes in the brain, including loss of vascular integrity, microthromboses, gliosis, demyelination, and neuronal- and glial injury and cell death, in both unvaccinated and vaccinated individuals irrespective of the severity of COVID-19. Recent data suggest that microglia play an important role in sustained COVID-19-related inflammation, which contributes to the etiology initiating a neurodegenerative cascade, to the worsening of pre-existing neurodegenerative disease or to the acceleration of neurodegenerative processes. Histopathological data have been supported by neuroimaging, and epidemiological studies also suggested a higher risk for neurodegenerative diseases after COVID-19.

**Conclusions:**

Due to the high prevalence of COVID-19 during the pandemic, healthcare systems should be aware of, and be prepared for a potential increase in the incidence of neurodegenerative diseases in the upcoming years. Strategies may include follow-up of well-described cohorts, analyses of outcomes in COVID-19-registries, nationwide surveillance programs using record-linkage of ICD-10 diagnoses, and comparing the incidence of neurodegenerative disorders in the post-pandemic periods to values of the pre-pandemic years. Awareness and active surveillance are particularly needed, because diverse clinical manifestations due to earlier SARS-CoV-2 infections may no longer be quoted as post-COVID-19 symptoms, and hence, increasing incidence of neurodegenerative pathologies at the community level may remain unnoticed.

## Introduction

Acute effects of SARS-CoV-2 infection on endothelial cells, glial cells, or neurons might progress to chronic damage in the brain tissue, resulting in clinical signs of neurodegenerative disorders in the long term. Healthcare systems should be aware of and be prepared for the late consequences of the acute brain tissue damage in COVID-19. Therefore, we highlight the need for surveillance of the possible increase in the incidence of neurodegenerative diseases at the population level in the upcoming years after the COVID-19 pandemic. After a historical overview of neurological consequences of former pandemics, we summarize the possible pathomechanisms of COVID-19-related brain damage, and present recent pathological and clinical findings supporting the association with inflammatory mechanisms and subsequent neurodegeneration. Finally, we provide arguments for the need for long-term surveillance programs and suggest possible approaches for such programs. 

### Historical background

The largest pandemic to date in the twenty-first century was caused by the SARS-CoV-2 coronavirus with over 775 million cases and 7 million fatalities between 2019 and 2023 worldwide [1]. Large pandemics occurred before: the Russian flu at the end of the nineteenth century causing 1 million deaths worldwide [2], presenting with neurological signs—like loss of smell, headache, and paroxysmal neuralgic pain—in some of the cases [3]. The “Spanish flu” caused by the H1 N1 type of the influenza virus affected about 500 million people with about 50 million deaths between 1918 and 1920 [4]. With an overlapping time period (1915–1926), the pandemic of “encephalitis lethargica”—also known as von Economo encephalitis [5], affected about a million people with about 150 000 fatal cases. Because of the overlapping time periods, the pandemics of Spanish flu and encephalitis lethargica were considered by many as a single disease entity caused by influenza virus [6, 7]. Hundred years later, the COVID-19 coronavirus pandemic, similarly to the past pandemics, has been associated with the emergence of diverse neurological symptoms and diseases, and the causal role of SARS-CoV-2 in many of these has been verified. In a historical review comparing the consequences of the Spanish flu/encephalitis lethargica and COVID-19, the importance of considering clinical severity of COVID-19 in subsequent neurodegeneration was emphasized [8].

### Neurodegeneration after viral infections

Viral infections are known to be associated with a broad range of neurological symptoms and subsequent neuroinflammation and neurodegeneration [9]. The late neurodegenerative process after encephalitis lethargica is well known, and was defined as “postencephalitic parkinsonism”, although the direct relationship between these two conditions is equivocal [10]. Pathological studies of post-mortem samples could not, however, prove any virus including influenza virus as a causative agent [11]. Therefore, the cause of encephalitis lethargica still remains unknown, and recently, an autoimmune mechanism has been suggested in its pathogenesis [12, 13]. Levine et al. [9] compared cases from two large biobanks, the FinnGen and the UK Biobank, and identified 45 virus associations with neurodegenerative disorders in the FinnGen biobank, and 22 of these were confirmed in samples of the UK biobank. The hazard ratio for the diagnosis of a neurodegenerative disorder was the highest in the first year after the viral infection, and several associations were found between viral infections and either Parkinson’s disease (PD) or dementia in the 1–5 year interval, and for dementia in the 5–15 year interval. The strongest association was found between viral encephalitis and Alzheimer’s disease (AD), and some associations persisted even at 15 years after the initial viral exposure. Due to this late effect, Levine et al. [9] emphasized that long-term monitoring for neurodegenerative disorders in post-COVID patients may be justified. COVID-19 has also been associated with the development of rapidly progressive dementia with an increased risk of an AD diagnosis within 360 days after initial COVID-19 diagnosis [14]. The association of COVID-19 with increased risk of AD has been suggested in a Mendelian randomization study as well [15]. However, there are several limitations of described studies: they are retrospective or cross-sectional; they lack imaging, CSF, or serum biomarker evaluations. Several other viral exposures were associated with parkinsonism, including influenza, herpes simplex, and hepatitis B and C virus [16], and the possible association with SARS-CoV-2 infection has also been suggested [17].

## Methodology

The NeuroCOVID-19 Task Force of the European Academy of Neurology has been engaged in several activities during the pandemic, and initiated numerous surveys and projects [18]. By the end of the pandemic, there were reports from histopathological studies on the possible association of SARS-CoV-2 infection with an increased risk of neurodegeneration. For this reason, the Task Force decided to evaluate the available evidence on this association. We performed a literature search of English language publications in MEDLINE with the search strategy:

((COVID-19[Title]) OR (SARS-CoV-2[Title])) AND ((neurodegen*[Title]) OR (dementia[Title]) OR (Alzheimer[Title]) OR (Parkinson[Title])). The last search in MEDLINE was run on April 20, 2024. The reference list of the identified articles has been checked for further relevant papers. Two authors (DB and AD) selected the articles for the first draft of the manuscript. The draft had been sent to all co-authors for comments in three rounds, and additional articles recommended by the members of the Task Force were also considered during the subsequent versions of this narrative review, until consensus was reached among all authors.

### Possible pathomechanisms in COVID-19-related long-term damage of the central nervous system

**Fig.1:**
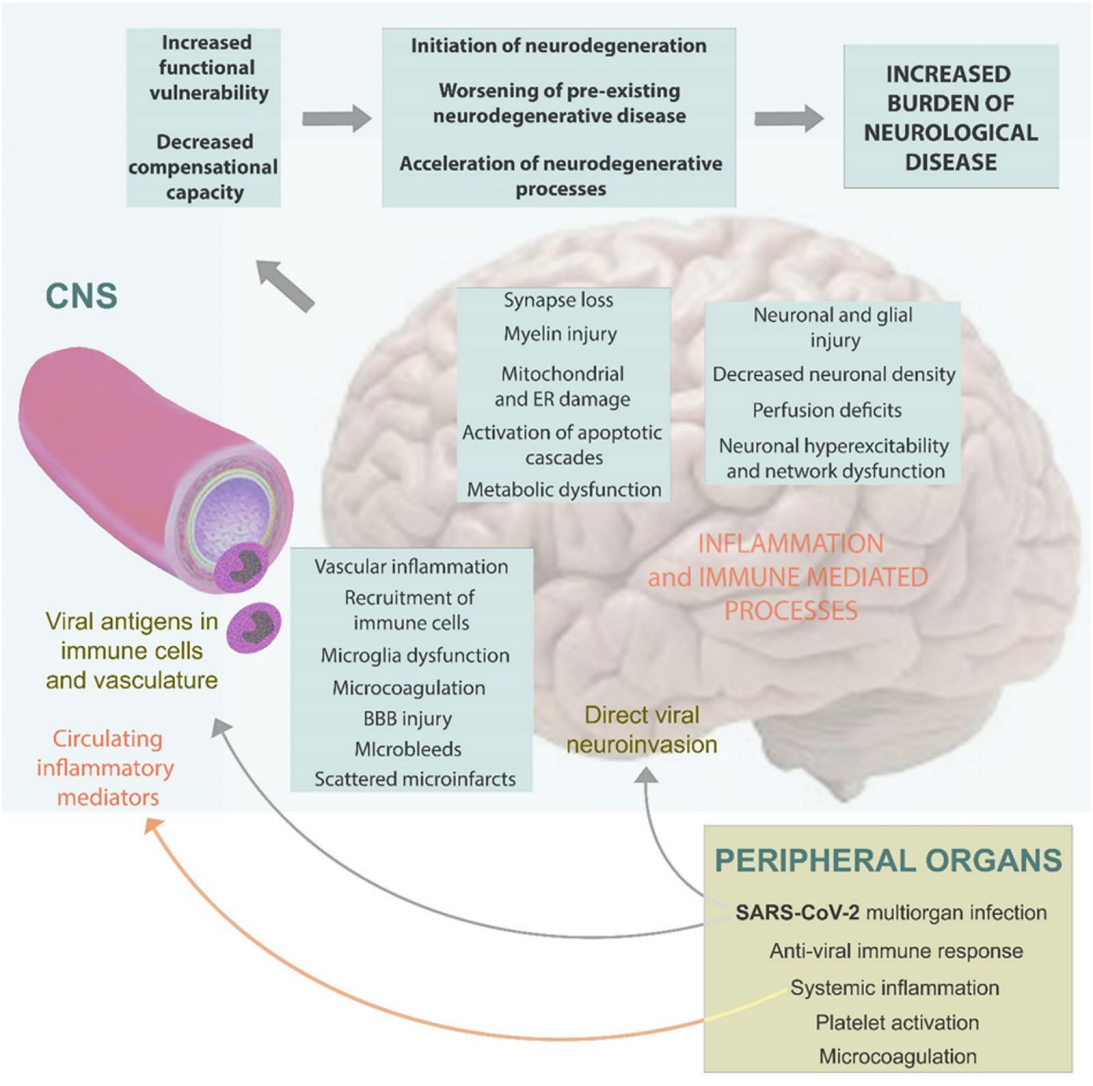
Possible pathomechanisms of COVID-19-related long-term damage of the central nervous system resulting in the increased incidence of neurodegerative diseases. *BBB* Blood–brain barrier; *CNS* Central nervous sytem; *ER* Endoplasmic reticulum

SARS-CoV-2 may cause neurodegeneration by different ways including direct neuroinvasion, neuroinflammation, and blood–brain barrier damage [19, 20] among several other mechanisms that are currently unexplored (Fig. 1).

#### Direct neuroinvasion

The role of direct viral invasion of the central nervous system (CNS) in SARS-CoV-2 infection is equivocal. Meinhardt et al. [21] suggested that SARS-CoV-2 virus enters the CNS via the olfactory transmucosal layer. On the other hand, the SARS-CoV-2 virus could be detected only in less than 3% of CSF samples (for review of 449 cases with neuro-COVID [22]). Data show that CSF RNA levels are generally very low [23]. Because RNA degradation may also contribute to low CNS RNA levels, lack of SARS-CoV-2 viral RNA positivity in the CSF in given cases does not necessarily mean that the infection is not present in vascular, ventricular, or parenchymal tissues. SARS-CoV-2 infection may persist in the brain parenchyma for months after the acute phase of the disease [24]. Giordano et al. [7] suggested that the infection might trigger neurodegeneration, starting in the olfactory bulb, in predisposed patients, and brought up the potential long-term neurological sequelae of the SARS-CoV-2 virus. Although the mechanism and the trajectories are different, altered smell sensation is a common sign of both COVID-19 and PD [25, 26], and suggests that the viral invasion of the olfactory system in COVID-19 might pave the way for CNS entry of the SARS-CoV-2 virus. Ageusia/dysgeusia is a common symptom of acute COVID-19. In neuropathological studies, viral proteins were detected in different brainstem nuclei, including the nucleus of solitary tract, responsible for taste function [27]. Toll-like receptor 2 (TLR2) with an important role in the pathogenesis of neurodegenerative disorders may have a critical role in the response to SARS-CoV-2 infiltration in the CNS [28], resulting in induction or acceleration of AD and PD pathologies.

#### Inflammatory and immune-mediated processes

It is considered that immune-mediated mechanisms are the key components in neuro-COVID for initiating the neurodegenerative cascade [22]. The review of Furman et al. [29] suggested that both COVID-19 and AD share pathological features and risk factors. The association between COVID-19 and AD may be explained by mutual risks and mechanisms [30]; with neuroinflammation being the major connection between COVID-19 and AD. COVID-19 may be associated with diverse clinical and imaging neurological abnormalities, which can predict poor outcome in some patients [31, 32]. The mechanisms underlying COVID-19-associated neurological symptoms that are highly heterogeneous in appearance, duration, and severity in individual patients are not well understood [33–35]. Of note, the severity of neurological symptoms does not seem to correlate well with the severity of SARS-CoV-2 infection in individual patients [36, 37].

From recent studies, it has become clear that neurovascular pathologies, coagulopathy, and marked changes in glial cells in multiple brain areas are associated with the development of central and systemic inflammation in COVID-19 patients [37–40].

Gliovascular pathological changes are highly heterogeneous in individual patients, occurring in multiple foci across the brain, including the medulla, hypothalamus, thalamus, and different sites of the cerebral cortex [23, 41–45], with most severe degenerative, inflammatory, and gliovascular pathologies in the medulla and the hypothalamus, and at central autonomic and neuroendocrine nuclei [23, 41, 42, 44, 46].

In post-mortem studies from deceased individuals at various stages of COVID-19, proteomic and transcriptomic profiling of brainstem, cerebellum, and olfactory tissues in early- and late-phase COVID-19 shows an inflammatory type I interferon response in acute COVID-19 cases, which resolves in the late disease phase. Using single-nucleus RNA sequencing and spatial transcriptomics, two patterns of reaction to severe systemic inflammation was noticed, one neuronal with a direct focus on cranial nerve nuclei and a separate diffuse pattern affecting the whole brainstem [47].

To study the processes of CNS damage in COVID-19, a novel autopsy platform allowing the collection of both fixed and frozen tissue mirror blocks (adjacent tissue blocks with identical orientation) from brain and peripheral organs of the same patients was introduced by the group of Ádám Dénes [23]. They found that microglia, the main inflammatory cell types of the CNS, show marked morphological transformation and dysfunction (loss of core microglial genes, metabolic failure, and mitochondrial damage) at sites of excessive synapse- and myelin phagocytosis and loss of glutamatergic terminals in COVID-19 brains at sites of severe vascular inflammation. Interleukin-1 and interleukin-6 are major proinflammatory cytokines linking central and systemic inflammatory profiles in COVID-19. Multiorgan (lung, spleen, and liver) viral load correlates with central viral load in given patients. In line with this, viral antigens are found perivascularly and in intravascular immune cells (monocytes, neutrophils, etc.), but little or no antigen positivity is observed in parenchymal neurons. Single-nucleus RNA sequencing, proteomics, analysis of inflammatory, and metabolic signatures revealed distinct mechanisms of microglial dysfunction associated with cerebral SARS-CoV-2 infection. Major metabolic failure and mitochondrial changes in glial cells and vascular elements at severely affected brain sites were also revealed [23], while neuronal pathologies appear secondary to these changes at these sites.

Supporting neuropathological findings, sustained systemic inflammation, and persistent localized BBB dysfunction is also involved in post-COVID cognitive symptoms [48]. In line with this, the SAVE-MORE trial showed that the interleukin-1 receptor antagonist anakinra guided by soluble urokinase plasminogen receptor plasma levels markedly reduced mortality, blood IL-6 and CRP levels and shortened hospital stay in COVID-19 patients [49]. Inflammatory changes associated with microglial dysfunction seen in multiple parts of the brain in COVID-19 cases may also provide implications concerning the recently identified role of microglia in controlling pathological network activity, cerebral blood flow, and hypoperfusion based on data from experimental studies [50–53]. In line with this, in COVID-19 patients, meta-analysis of EEG findings shows frequent abnormal background activity, whereas long-term microstructural and cerebral blood flow changes were observed in patients recovered from COVID-19 correlated with CRP and IL-6 levels even in the absence of neurological manifestations [54, 55].

#### Microcoagulation, microbleeds, and other processes

COVID-19 is not only associated with an increased risk of stroke, but systemic inflammatory mechanisms that influence outcome in stroke have been raised to show similarities to those seen in COVID-19 [56]. These include, but are not limited to changes in BBB function, initiation of procoagulatory cascades, neuronal excitotoxicity, cerebral perfusion defects, emergence of pathological neuronal activity patterns, and higher levels of neurotoxicity due to vascular leakage of plasma proteins, recruitment of blood-borne immune cells, or glial dysfunction. A possible role of microbleeds was reported by Mitra et al. [57] and confirmed by a meta-analysis of Boparai et al. [58] who reported MRI abnormalities in 55% of COVID patients, with 20% of all patients presenting microbleeds. Most studies included in this meta-analysis were published in 2020–2021, when the most severe variants persisted. Microbleeds however, can be the consequence of mechanical ventilation in COVID-19 patients, as well [59].

Dysfunction of the mitochondria [60] or endoplasmic reticulum may also play a role [61] considering that the virus may hijack these systems to sustain the reproduction of the new virions. CSF and serum biomarkers of amyloid processing were compared in a small case–control study of patients with COVID-19 neurological syndromes, raising the possibility of impaired amyloid processing in COVID-19 [62]. Based on pre- and post-infection neuroimaging data, SARS-CoV-2 is associated with changes in brain structure, including reduced brain size and gray matter thickness that may persist for months after the onset of SARS-CoV-2 infection [63].

### Clinical data supporting the association of SARS-CoV-2 infection and neurodegenerative diseases

The association of COVID-19 with subsequent neurodegenerative disorders was reported in clinical observations, and in analyses of healthcare administrative databases.

#### Clinical observations on cognition and related effects

In a large population-based survey data of over 110 thousand subjects, Hampshire et al. [64] reported that infection with the original virus or the alpha variant, longer illness duration, and hospitalization due to COVID-19 were predictors of more severe cognitive deficits. Short-duration COVID-19 was also associated with detectable cognitive deficits after recovery. Hampshire et al. emphasized that longer-term persistence of cognitive deficits and their clinical relevance are currently unclear, and surveillance is needed in the future. In this large community-based study, COVID-19 was associated with longer-term, objectively measurable cognitive deficits that may persist for a year or more after the acute infection [64]. These data should be interpreted cautiously also considering that the pandemic-related isolation and other measures impacted on the mental health of the population in general and cognitive performance of elderly people more in particular.

A meta-analysis of six studies with 175 patients at least 3 weeks after the diagnosis of COVID-19 and 275 healthy individuals Sobrino-Relano et al. [65] found that individuals who had recovered from COVID-19 showed significant cognitive deficits compared to controls. A further systematic review of 7912 COVID-19 survivors two years after the infection reported cognitive impairment in 27.6% of the cases [66]. Finally, a systematic review of 12 cohort studies involving 2.7 million post-COVID cases and 30 million controls found a 50% increased risk for newly diagnosed AD, and 44% increase in the risk of newly diagnosed PD at 3–24 months after recovering from COVID-19. The authors suggested developing a comprehensive surveillance program for neurodegenerative diseases in recovered COVID-19 patients [67]. It should be noted that several confounding factors could not be considered in this meta-analysis, and some patients might have already suffered from AD or PD, which was unmasked or identified when these persons sought for medical help due to COVID-19. In addition, making new diagnoses of PD and AD was delayed due to the reduced availability of health services during pandemic. Instead of being gradually diagnosed at a regular rate, all the accumulated cases may have come up for diagnosis once the health services were restored. This is one reason why continuous surveillance is needed—to check whether numbers return to usual or remain elevated.

The systematic review and meta-analysis of the cognitive effects of COVID-19 in adults with no prior history of cognitive impairment demonstrated that patients recovered from COVID-19 had impaired executive functions, attention, and memory and post-COVID-19 patients have lower general cognition compared to healthy controls up to 7 months post-infection [68].

#### Analyses of healthcare administrative databases

Electronic health records provide the option of evaluating a large number of subjects over time, and by record-linkage associations can be found between COVID-19 and subsequent conditions, including neurodegenerative diseases. A British study with relatively short-term follow-up (6 months after COVID-19) reported that one-third of 236 thousand patients diagnosed with COVID-19 had a neurological or psychiatric diagnosis, and of the total number 12.8% received their first such diagnosis at this time [69]. The rate of these newly diagnosed conditions—including PD and dementia—was significantly higher in those who needed intensive care during COVID-19: 0.26% vs 0.11% for PD and 1.74% vs. 0.67% for dementia. There is, however, a potential bias that some of these people might have had a pre-existing condition, which was diagnosed as such after the patient sought medical help for COVID-19. The risks of some neurological and psychiatric sequelae—possibly including post-traumatic stress disorders—in patients hospitalized with COVID-19 have a wide range and are long standing for at least two years [70, 71]. In an analysis of over 6 million elderly adults with COVID-19, there was a significantly higher risk for new diagnosis of AD in the first year after the initial COVID-19 diagnosis [14]. In these studies, it cannot be excluded that the already pre-existing conditions were diagnosed in at least some of the patients.

In a study from Denmark, electronic healthcare records covering half of the total population were analyzed for the association of COVID-19 and neurodegenerative diseases for a less than 2 year period. Among the 43,375 patients tested SARS-CoV-2 positive (35,362 outpatients, 8,013 inpatients), the risk of AD was 3.5-fold and 3.4-fold at 6 and 12 months later, whereas the risk for PD was 2.4-fold and 2.2-fold higher at 6 and 12 months compared those who had negative test results for COVID-19 [72]. Among outpatients, the risk of neurodegenerative and cerebrovascular disorders was increased among COVID-19-positive compared to COVID-negative outpatients. For patients hospitalized for COVID-19, this increased risk was similar compared to hospitalized patients with influenza or bacterial pneumonia.

All these clinical data refer to the relatively short-term period after COVID-19 and are likely biased. The evaluation of late consequences remains a challenging task for the future. To establish the long-term consequences of COVID-19 there are tasks remaining for both experimental/pathological studies and clinical observations.

### Remaining tasks for neuropathology and clinical imaging studies

Because present data remain highly controversial regarding the extent of productive SARS-CoV-2 infection in the brain parenchyma and in the CSF, post-mortem studies of COVID-19 cases need to perform comprehensive comparison of CSF and brain tissue virus mRNA levels to assess the incidence of central virus load.

Gliovascular and inflammatory mechanisms in COVID-19 have to be understood in detail. To this end, further neuropathological studies are required. It may be difficult to find appropriate “control cases” given that the vast majority of the population has been infected/vaccinated. In this regard, it is important to perform cross-comparison of pre-pandemic and post-pandemic neuropathology materials not only concerning controls for COVID-19 tissues, but also to assess any additive effects of COVID-19 to the progression of neurodegeneration in other disorders.

It is also important to correlate focal sites of brain inflammation with neurological symptoms in neuropathological studies (retrospective analysis of clinical symptoms plus neuroimaging data).

In post-COVID cases, neurological symptoms need to be correlated with imaging of brain inflammation (e.g., translocator protein total distribution volume—TSPO VT PET), metabolism (PET), cerebral perfusion/functional connectivity (MRI, EEG), and blood biomarkers. Some relevant studies have already been initiated. For example, available TSPO-PET data suggest lasting inflammatory changes in post-COVID-19 cases and some correlation between focal signal intensity changes and neurological function (e.g., TSPO VT in the dorsal putamen of COVID-19 cases negatively correlated with motor speed [73]).

An important open point is to study whether vaccination and virostatic therapy have a protective effect against COVID-19-related neuropathological changes and neurological sequelae.

The remarkable association between systemic inflammatory burden and clinical (including neurological) outcome in COVID-19 patients calls for the need to study the mechanisms revealed in other disorders to gain further insight into the means how central and systemic inflammatory mechanisms could contribute to diverse COVID-19-related neuropathologies.

Corresponding to neuropathological findings, imaging studies also show diverse abnormalities in multiple brain areas, including the cerebral cortex, striatum, medulla, and thalamus. Changes include focal hyperintensities on MRI, loss of vascular integrity, microcoagulations, gliosis, demyelination, neuronal and glial injuries, and cell death [21, 39, 74–78]. Correlative neuroimaging and neuropathological studies in the years after the acute SARS-CoV-2 infection should be performed to identify the localization and the extent of late changes attributable to COVID-19, detectable by complementary imaging techniques.

Although in the short term, PD diagnosis soon after COVID-19 is rare, long-term re-evaluation is recommended [79].

As the pandemic occurred in an aging population, the possible additive effect of increasing age and the former SARS-CoV-2 infection should also be considered as explanatory factors of an increased incidence of neurodegenerative diseases [8]. For this reason, a difference may be expected between populations with different aging patterns.

Most COVID-19 registries have recorded the presence of neurodegenerative diseases at patient entry and follow-up, like the ENERGY registry [33, 80], the Spanish SEMI-COVID-19 Registry [81], the German LEOSS registry [82], and the Eurasian international ACTIV SARS-CoV-2 registry [83] to evaluate chronic non-infectious diseases dynamics after COVID-19 infection in adult patients. A long-term follow-up of patients in these registries should be performed to identify new cases of neurodegenerative diseases and their correlates.

A recent systematic review of 126 studies and over 1.5 million subjects with COVID-19 found that 12 months after the acute infection, the prevalence of cognitive impairment was close to 15% higher than the pre-COVID values [84]. Systematic reviews of long-term follow-up studies needed to be performed and regularly updated in the future.

Longitudinal studies based on biomarkers (CSF, serum, and PET-CT) are needed to assess direct link between neuroinflammation triggered by SARS-CoV-2 and neurodegenerative disorders.

### Need for long-term surveillance programs for neurodegenerative disorders after the COVID-19 pandemic

Due to the large number of subjects affected by the SARS-CoV-2 infection, the CNS effects of the acute infection and the possibility of late consequences several years after the viral exposure need to be explored. A number of studies already concluded that awareness for the possibility of the development of neurodegenerative diseases and long-term surveillance of the possible increase in the incidence of neurodegenerative diseases at the population level is justified [7, 13, 14, 30, 67, 72, 79, 85]. The most important arguments are as follows:

1. Infections by different viruses are associated with the development of neurodegenerative diseases as late as 15 years after the initial viral exposure, and the strongest association was detected between encephalitis and dementia [9].
2. There is firm evidence from neuropathological studies that SARS-CoV-2 infection in the acute phase results in severe inflammatory changes throughout the CNS (vascular inflammation, microglial dysfunction, metabolic failure in gliovascular compartments, changes in blood–brain barrier integrity, excessive synapse and myelin loss, etc.). Neuropathological features are also present in patients infected with delta and omicron variants, although extensive comparative studies with earlier, more pathogenic variants remain to be performed [86, 87].
3. Loss of neurons due to the acute inflammatory changes may result in the chronic post-COVID condition in higher incidence of neurodegenerative diseases by several ways. These processes may relate to the development of neurodegenerative diseases by possible contribution to their etiology (induction), by worsening the already existing disease (superposition) or by accelerating the neurodegenerative processes thus resulting in earlier manifestation (anticipation) of the clinical signs [7, 8, 30].
4. Awareness for the possible increase in the incidence of neurodegenerative diseases is needed due to the considerable portion of the society affected by SARS-CoV-2 exposure, as even a small increase in the incidence of neurodegenerative diseases would affect a large number of subjects [88].
5. Annual survey of national electronic administrative health care records covering a large part of the population could identify an increase in neurodegenerative diseases at the population level [89].

### Methods suggested for surveillance

Surveillance may be performed both at individual and population levels. Several COVID registries involving patients with neurological issues related to the virus exist in the world (for example, Frontera et al. [90]; Beghi et al. [91]). The follow-up of patients in such registries might provide valuable data on detailed aspects of the acute infection related to later development of neurodegenerative diseases. The advantage of this approach would be the availability of detailed individual data allowing finding associations between several patient characteristics with long-term outcome. On the other hand, funding and patient tracking in these registries would be a challenge.

At the population level, healthcare administrative databases may be used for long-term evaluations, with record linkage to identify those with and without records of previous COVID-19 during the pandemic. The advantage of this approach is the involvement of millions of subjects, thus providing more meaningful data for defined populations. Using such data, however, it is impossible to fully differentiate individuals with and without COVID-19 during the pandemic, as many of those not having COVID-19 records might also have had the disease. In many countries, it was precisely documented if a person was vaccinated. This data source should also be considered. To analyze such large databases informatic methods using artificial intelligence could be used [92]. The use of administrative healthcare records does not allow considering those with asymptomatic or unrecognized COVID-19 during the period of the pandemic, especially at the later stages/years. For these reasons, neither case identification nor case certification will be accurate with this approach.

However, there is another approach to use administrative health care records to detect late consequences of COVID-19. Instead of comparing those with and without records of SARS-CoV-2 infection in the pandemic period, incidence of neurodegenerative diseases can be compared for periods before and after the pandemic. For such analyses, the basis period for comparison could be any year up until 2019. In such evaluations, multivariate analyses should be applied to consider such confounders that may have also changed between the pre-COVID basis year and the post-COVID period of interest, like the increasingly aging population, the level of exposition to pollution/pesticides, which fluctuates with the industrialization of the country as well as food and drug regulations.

In summary, the following approaches may be used to identify an increase in neurodegenerative diseases associated with previous infection with SARS-CoV-2:

- Follow-up of individual patients in COVID registries and of patient cohorts. Some registries or patient cohorts provide high-level data for studying pathological changes. These should be supported by grants to proceed with follow-up and understand potential disease mechanisms.
- Record linkage in administrative health care databases comparing the appearance of new neurodegenerative disease diagnoses in those with and in those without documented COVID-19. However, due to the high prevalence of SARS-CoV-2 infection, and the less severe or even asymptomatic disease at the later stage of the pandemic, it will be difficult to identify reliably those who did not have SARS-CoV-2 infection.
- Comparing neurodegenerative disease incidence to values in the population in the pre-COVID era (up till 2019) using administrative health care records.

## Conclusions

Neurological manifestations after COVID-19 may be multifaceted and long-lasting. The associations of the neuropathological changes in the acute phase with subsequent neurodegeneration are highly likely and the role of inflammation as an underlying mechanism is emerging. An increase in the incidence of neurodegenerative diseases might be expected. Awareness is particularly needed given that clinical manifestations months or years after SARS-CoV-2 infections may no longer be quoted as post-COVID symptoms, and hence, progressing pathologies at the community level may remain unnoticed. 

## Data Availability

Not applicable.
